# Sevelamer Carbonate Crystal-Induced Colitis

**DOI:** 10.1155/2020/4646732

**Published:** 2020-07-23

**Authors:** T. Lai, A. Frugoli, B. Barrows, M. Salehpour

**Affiliations:** ^1^Community Memorial Health System, Graduate Medical Education, Ventura, CA, USA; ^2^Community Memorial Health System, Graduate Medical Education, Department of Internal Medicine, Pacific Inpatient Physicians, Ventura, CA, USA; ^3^Community Memorial Hospital, Department of Pathology, Ventura, CA, USA; ^4^Community Memorial Hospital, Department of General Surgery, Ventura, CA, USA

## Abstract

Hyperphosphatemia is a common and well-described complication of end-stage renal disease. Despite strict dietary constraints and compliance, phosphate binders such as calcium acetate and/or sevelamer carbonate are also needed to treat secondary hyperparathyroidism. This case vignette describes an underrecognized adverse effect of a phosphate binder, sevelamer carbonate, inducing colitis in a 47-year-old male with insulin-dependent diabetes complicated by end-stage renal disease. He presented for recurrent abdominal pain with associated nausea and was found to have multiple circumferential lesions on computed tomography including distal ascending, transverse, and proximal descending colon. Colonoscopy demonstrated nearly obstructing lesions worrisome for colonic ischemia or inflammatory bowel disease. Pathological review of histology demonstrated ragged colonic mucosa with ulcerative debris and nonpolarizing crystalline material at the sites of ulceration, morphologically consistent with the phosphate binder, sevelamer carbonate. Sevelamer carbonate was discontinued, and the patient was transitioned to calcium carbonate with strict dietary restrictions. His symptoms improved with the cessation of sevelamer, and he was subsequently discharged home. He eventually underwent renal transplant without redevelopment of symptoms. Recognition of this underreported complication of sevelamer carbonate, phosphate binder, is of utmost importance in directing appropriate therapy with cessation of this medication in the setting of gastrointestinal complaints or more specifically enteritis and colitis. Clinicians providing care to end-stage renal patients taking either sevelamer and/or sodium polystyrene sulfonate should have increased awareness of the possible gastrointestinal side effects.

## 1. Introduction

End-stage renal disease (ESRD) is associated with multiple metabolic and electrolyte derangements. Hormonal and electrolyte imbalance of calcium, phosphorus, and PTH can result in short-term complications such as calciphylaxis and long-term sequelae such as renal osteodystrophy, increased cardiovascular events, and all-cause mortality [1–3]. As a result, many patients have strict dietary constraints, and despite compliance, phosphate binders such as calcium acetate and/or sevelamer carbonate are also needed to treat secondary hyperparathyroidism [1, 2]. Of the two more commonly used, sevelamer carbonate is a calcium-free phosphate binder that is composed of a nonabsorbable resin and has been considered one of the preferred agents by NKF KDOQI guidelines [4, 5]. It has many common gastrointestinal side effects including nausea, vomiting, constipation, and abdominal pain that limit patient compliance [2, 6]. These symptoms previously had no specific etiology, but recently, sevelamer carbonate is receiving recognition as a possible cause of mucosal injury [6–8]. The exact mechanism of injury remains elusive, but in the last 5 years, Swanson et al. described that variable degrees of mucosal injury throughout the gastrointestinal tract from “gum to bum” are believed to be associated with sevelamer use. These include acute and chronic inflammation, ulceration, and features of ischemia with mucosal necrosis from samples obtained via endoscopy/colonoscopy [7].

## 2. Clinical Case

A 47-year-old male with hypertension and insulin-dependent diabetes complicated by end-stage renal disease presented with a 2-day history of crampy abdominal pain. It was associated with nausea and anorexia but without additional gastrointestinal symptoms such as diarrhea or hematochezia. He had a similar episode 1 month prior with workup including computed tomography imaging followed by colonoscopy, notable for numerous circumferential lesions.

**Figure 1:**
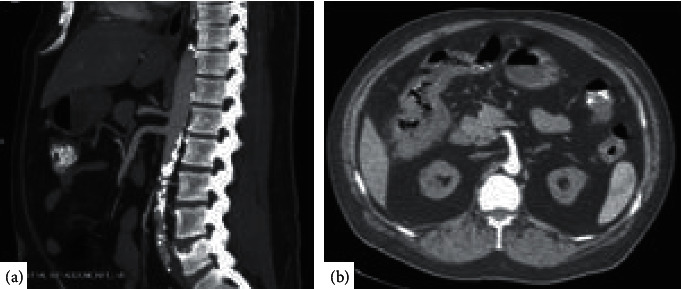
Computed tomography angiogram with contrast of the abdomen and pelvis demonstrating atherosclerotic disease of the abdominal aorta without involvement of major arterial supply of the intestine (a) and pericolic fat stranding with wall thickening of the right hepatic flexure (b).

**Figure 2:**
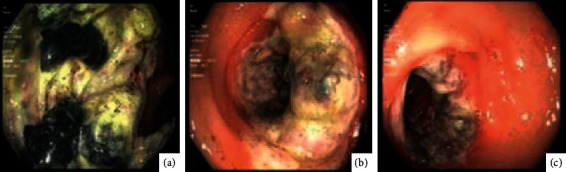
Images from colonoscopy demonstrating multiple lesions including obstructing circumferential lesion in the ascending colon (a, b) and circumferential but nonobstructing lesion in the transverse colon (c).

**Figure 3:**
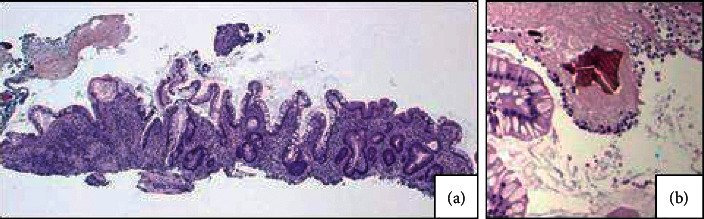
(a) Ragged colonic mucosa from the circumferential lesions biopsied during colonoscopy. (b) A high power view of a sevelamer crystal on hematoxylin and eosin-stained (H&E) slide with characteristic “fish scale” morphology and yellow/gold color. Both sevelamer (Figure 3(b)) and sodium polystyrene sulfonate have similar “fish scale” morphology and are nonpolarizable by polarized light microscopy. The primary morphologic difference between these two crystal types can be seen on standard H&E-stained slides. Sevelamer crystals can be distinguished by yellow/gold color when compared with sodium polystyrene sulfonate crystals, which show dark violet staining.

**Figure 4:**
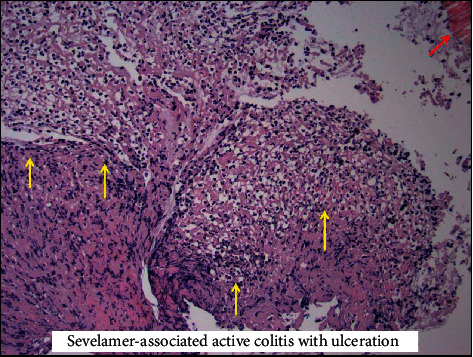
Image from histology demonstrating ulcerated colonic mucosa with adherent fibrinous exudate (yellow arrows) in the presence of sevelamer crystals (red arrow) seen in the top right corner.

**Figure 5:**
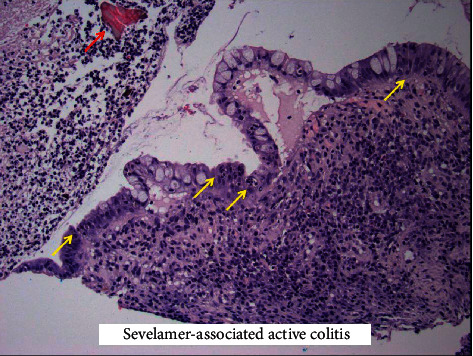
Image from histology demonstrating active colitis (yellow arrows) again in the presence of sevelamer crystals (red arrow) as previously seen, top left corner.

Biopsy histology was suspicious for colonic ischemia, and there were no histologic features of inflammatory bowel disease. It was suspected this constellation of symptoms could have developed as sequelae to hypotension during hemodialysis while on antihypertensive medications. His antihypertensive medications were discontinued, and he was provided supportive care with nonoperative management. He had resolution of his pain and was able to tolerate diet and was discharged home. He returned one month later for recurrence of abdominal pain and nausea. He was empirically started on antimicrobials and underwent repeat imaging with the computed tomography mesenteric angiogram that demonstrated only moderate features of atherosclerotic disease with patent celiac, superior mesenteric artery (SMA), and inferior mesenteric artery (IMA) (Figure 1(a)). Additional findings included features of colitis with pericolic fat stranding in the distal ascending, transverse, and proximal descending colon (Figure 1(b)). He underwent repeat colonoscopy showing similar findings (3 regions of circumferential ulceration in the ascending/hepatic flexure, splenic flexure, and descending colon) (Figures 2(a)–2(c)). Biopsy histology demonstrated ragged colonic mucosa with ulcerative debris and nonpolarizing crystalline material at the sites of ulceration (Figures 3–5). Clinical workup for additional causes including cardioembolic events and vasculitis was completed and found to be nondiagnostic. Histology from the current and prior colon biopsy specimens demonstrated crystalline material morphologically consistent with the phosphate binder sevelamer carbonate. Sevelamer carbonate was discontinued, and the patient was transitioned to calcium carbonate with strict dietary restrictions. His symptoms improved with the cessation of sevelamer, and he was subsequently discharged home. He eventually underwent renal transplant without redevelopment of symptoms.

## 3. Discussion

This vignette illustrates a rare and likely underrecognized adverse effect of therapy with sevelamer carbonate in the treatment of hyperphosphatemia in patients with end-stage renal disease. Review of the literature shows a limited number of reported cases (estimate of about 50 cases) and illustrates the immense range of presenting symptoms and possible pathology identified. This condition is likely more common, but underrecognized as there are many reasons for patients with end-stage renal disease to have gastrointestinal complaints. Additionally, the relative rarity of complications with severity to prompt further evaluation is complicated by nonspecific colonoscopic findings and potentially subtle histopathologic features, which may not include crystals dependent on biopsy sampling. It is also possible that the pathology is missed as a result of misidentification or failure to identify crystals from resin-based medications like sevelamer and sodium polystyrene sulfonate [9–13].

Although not previously described, there are clinical circumstances when patients with ESRD may chronically be taking more than one medication associated with mucosal injury. This creates a diagnostic dilemma, in which the diagnosis may solely rely on the histopathologic review of tissue and identification by crystal morphology. As previously discussed by Gonzales et al. in their review and comparison of classic features of medication resins, sevelamer carbonate morphology was described as flaky (commonly described as “fish scales”), rectangular in shape, with hues of pink (central) with peripheral transition to yellow/orange pigment by H&E staining [9].

Recognition of this underreported complication of sevelamer carbonate, phosphate binder, is of utmost importance in directing appropriate therapy with cessation of this medication in the setting of gastrointestinal complaints or more specifically enteritis and colitis. Clinicians providing care to end-stage renal patients taking either sevelamer and/or sodium polystyrene sulfonate should have increased awareness of the possible gastrointestinal side effects.
